# miR-450b-5p loss mediated KIF26B activation promoted hepatocellular carcinoma progression by activating PI3K/AKT pathway

**DOI:** 10.1186/s12935-019-0923-x

**Published:** 2019-07-31

**Authors:** Hua Li, Shen Shen, Xiaolong Chen, Zhigang Ren, Zhiqin Li, Zujiang Yu

**Affiliations:** 1 Department of Infectious Diseases, The First Affiliated Hospital of Zhengzhou University, 1# Jianshe East Road, Zhengzhou, 450052 China; 2 Gene Hospital of Henan Province, Precision Medicine Center, The First Affiliated Hospital of Zhengzhou University, Zhengzhou, 450052 China

**Keywords:** Kinesin family member 26B (KIF26B), Hepatocellular carcinoma (HCC), miR-450b-5p, PI3K/AKT signal pathway

## Abstract

**Background:**

Kinesin family member 26B (KIF26B) is unveiled acted as important role in many solid tumors, however, the function of KIF26B in hepatocellular carcinoma (HCC) is unclear.

**Methods:**

The expression of KIF26B in HCC tissues and cell lines were measured with immunochemistry, real-time PCR and western blotting. The correlation between KIF26B expression and clinicopathological characteristics were analyzed by SPSS19.0. Functional experiments of KIF26B was conducted by CCK-8, transwell, EDU, colony formation in vitro and tumorigenesis in vivo. The gene set enrichment analysis was used to search the downstream pathway, luciferase reporter experiment was used to find the upstream regulatory factor of KIF26B.

**Results:**

In this study, we found that KIF26B was overexpressed both in HCC tissues and cell lines. High expression of KIF26B was associated with poor overall survival (OS), late TNM stage and poor differentiation. Loss of function experiments showed that suppression of KIF26B could inhibit cell viability, proliferation rate and invasion ability of HCC cells. KEGG and GO analysis showed that expression of KIF26B was highly relevant with PI3K/AKT signal pathway, and suppression of KIF26B could decrease the expression of m-TOR, p-PI3K and p-AKT. Further study demonstrated that expression of KIF26B was negative correlated with miR-450b-5p level in HCC tissues, and miR-450b-5p could inhibit cell viability, proliferation rate and invasion ability of HCC cells via targeted inhibiting KIF26B.

**Conclusion:**

Our study demonstrated that miR-450-5p/KIF26B/AKT axis is critical for progression of HCC, and might provide novel prognostic biomarker and therapeutic target for HCC.

**Electronic supplementary material:**

The online version of this article (10.1186/s12935-019-0923-x) contains supplementary material, which is available to authorized users.

## Background

Hepatocellular carcinoma is a common digestive system tumor with biological characteristics of high invasiveness and high mortality [1]. At present, the comprehensive treatment plan based on surgical resection and chemotherapy embolization is a great improvement in the prognosis of patients with HCC. However, most of the patients are diagnosed in the middle and late stage due to lack of effective early diagnostic biomarkers, and have poor prognosis despite treatment [2, 3]. Therefore, it is urgent and important to investigate novel biomarkers to improve the rate of early diagnosis of HCC.

The human KIF26B, a member of the kinesin (KIF) family, is known for its function on adhesion and polarization of mesenchymal cells [4]. Recent studies showed that overexpression of KIF26B was associated with poor prognosis of solid tumors including breast cancer [5, 6], gastric cancer [7] and colorectal cancer [8]. In addition, Pu et al. found that KIF26B was essential for multi-drug resistance in osteosarcoma [9]. However, no studies reported the biological role of KIF26B in HCC. In this study, we found KIF26B was overexpressed in HCC tissues and cell lines. Overexpression of KIF26B was associated with poor overall survival (OS), late TNM stage and poor differentiation. Suppression of KIF26B could inhibit cell viability, proliferation rate, invasion ability and tumor formation ability of HCC cells, and such phenomenon might be correlated with inhibition of PI3K/AKT signaling pathway. Furthermore, we proved that KIF26b was a target gene of miR-450b-5p in HCC cells. Taken together, our study found that miR-450b-5p/KIF26B/AKT axis is critical for tumorigenesis and progression of HCC, such discovery may provide a potential therapeutic target for HCC treatment.

## Materials and methods

### Patients and specimens

Tissue microarrays (TMA) containing 93 paired paraffin embedding HCC specimen and corresponding non-tumor tissues was obtained from Shanghai Core Super Biotechnology Co., Ltd. All these patients were available follow-up data. A total of 369 HCC patients and 50 normal patients, with follow-up data, from The Cancer Genome Atlas (TCGA, https://tcga-data.nci.nih.gov/tcga/) database, were used for gene expression analysis and patients’ survival analyses according to our previous protocol [10].

### Cell culture

Normal (L02) and HCC cell lines (HepG2, SMMC-7721, Hep-3B and HCC-LM3) were purchased from ATCC (Manassas, USA) or Sibcb (Shanghai, China). Cells were cultured in DMEM supplied with 10% fatal bovine serum, and incubated in a humidified atmosphere of 95% air and 5% CO_2_ at 37 °C.

### Western blot

Protein isolation and western blotting have been described before [11]. Cells were collected at 48 h after transfection. Samples were probed with KIF26B, m-TOR, p-PI3K, p-AKT or GAPDH monoclonal antibody. Goat anti-mouse HRP antibodies were obtained from Zhongshan Jinqiao Company, Beijing. ECL detection system (Millipore, Bedford, MA, USA) is used to assess proteins expression.

### Total RNA isolation and quantitative real-time PCR (qPCR)

Total RNA from HCC cells was extracted using Trizol reagent (Invitrogen, Carlsbad, CA, USA) according to the standard RNA isolation protocol. Then reverse-transcribed with TransScript First-Strand cDNA Synthesis SuperMix (TransGen, Beijing, China). Quantitative real-time PCR (qRT-PCR) was performed using TransScript Top Green qPCR SuperMix (TransGen, Beijing, China) on 7500 sequence detection system (Applied Biosystems, Foster City, CA, USA). GAPDH was used as the endogenous controls. Data were analyzed using the comparative Ct method (2^−ΔΔCt^). Each group included three repeated wells.

### Immunohistochemistry

The Tissue microarrays (TMA) was rehydrated in a series of graded alcohol dilutions. Then heat epitope retrieval for 20 min in condition of Citrate salt solution. After closed by 5% BSA for 30 min, all tissues were incubated with a rabbit antibody to human anti-KIF26B, anti-Ki67, anti-m-TOR, anti-p-AKT and anti-p-PI3K overnight at 4 °C. Slides were then incubated with HRP at room temperature for 30 min and were visualized using DAB as chromogen for 5–10 min. The method for IHC score was according to the staining intensity score of 1–5: High KIF26B expression was defined as a staining score with 4–5, while low expression was defined as a staining score with 1–3.

### Transfection of Hep-3B and HCC-LM3 cell lines

Human-specific shKIF26B (1–3) and negative control were obtained from Shanghai Genechem Co., Ltd. and used to generate stable KIF26B knockdown cell lines according to the instructions of manufacturer. The coding sequences of human KIF26B and negative control were amplified and cloned into pcDNA3.1 (+) to generate KIF26B overexpression and according negative control (GenePharma, Shanghai, China). miR-450b-5p mimics, inhibitors as well as respective negative controls (NC) were commercially purchased from Gene Pharma (Shanghai, China). The cell transfection was conducted according to the instruction manual.

### Cell viability

Hep-3B and HCC-LM3 cell lines were transfected with KIF26B shRNA, Negative control and blank (or miR-450b-5p mimics, miR-450b-5p mimics and KIF26B and negative control). After 24 h, these cells were seeded into 96-well microplates with 5000 cells per well. Cell counting kit-8 (CCK-8) assay (Dojindo, Kumamoto, Japan) was then performed to detect cell viability in 24 h, 48 h and 72 h. The OD450 value was determined by using a MRX II microplate reader (Dynex, Chantilly, VA, USA).

### Transmembrane invasion assay

Human Hep-3B and HCC-LM3 cells were transfected with KIF26B shRNA and negative control (or miR-450b-5p mimics, miR-450b-5p mimics and KIF26B and negative control). After 48 h, 5 × 10^6^/mL cells were transferred into the upper chamber of the Millicell inserts pre-coated with 20 μg Matrigel and pre-incubated 1 h to reconstitute a basement membrane (Millipore, USA) in a serum-free DMEM. DMEM containing 10% fetal bovine serum was added to the lower chamber. After 24 h incubation, the cells that invaded through the membrane were fixed with methanol and stained with 0.5% crystal violet.

### EDU assay

Proliferating HCC cell lines (Hep3B and HCC-LM3) were determined by using the Click-iTEdU Imaging Kit (Invitrogen; Carlsbad, CA, USA) according to the manufacturer’s protocol. Briefly, cells were transfected with KIF26B shRNA and negative control (or miR-450b-5p mimics, miR-450b-5p mimics and KIF26B and negative control). After 48 h, 10 μM EdU for 2 h before fixation, permeabilization, and EdU staining. Cell nuclei were stained with Hoechst 33342 (Invitrogen) at a concentration of 5 μg/mL for 30 min.

### Colony-forming assay

Hep-3B and HCC-LM3 were transfected with KIF26B shRNA and negative control (or miR-450b-5p mimics, miR-450b-5p mimics and KIF26B and negative control), then they were dissociated with trypsin, resuspended in DMEM complete medium with 10% FBS, and inoculated into a 6-well plate at a density of 2000 cells/well. After 12 days, colonies were washed twice with PBS, dyed with crystal violet and photographed.

### Luciferase activity assay

The 3′UTR (3′ untranslated region) sequence of KIF26B containing the predicted miR-450b-5p-binding site and according the mutant sequence were subcloned into psiCHECK-2 luciferase reporter vector (Promega, Madison, WI, USA). The luciferase activity experiment was conducted according to the introduction manual.

### In vivo tumorigenesis assay

All experimental procedures involving animals were in accordance with the Guide for the Care and Use of Laboratory Animals and were performed as described previously [12]. The study protocol was also approved by the Committee on the Use of Live Animals in Teaching and Research, the First Affiliated Hospital of Zhengzhou University. For mice xenograft tumor model, 6- to 8-weeks-old male mice were used. HCC-LM3 cells transfected with shKIF26B or negative control were subcutaneously (s.c.) injected into the lower flank of the mice with 3.0 × 10^6^. The tumor volume was calculated by the formula: Volume = (width)2 × length/2. Weeks 1, 2, and 3 after injection, mice were photographed with an IVIS@ Lumina II system (Caliper Life Sciences, Hopkinton, MA) 10 min after an intraperitoneal injection of 4.0 mg of luciferin (Gold Biotechnology, Inc., St. Louis, MO) in 50 μL of saline. After 3 weeks, tumors were surgically removed and weighed.

### The gene set enrichment analysis (GSEA)

GSEA was used to determine which pathway was associated with KIF26B expression in TCGA data set. The expression profiles of 377 samples from TCGA data set was grouped two groups (KIF26B high and KIF26B low). GSEA v2.0 was used to determine whether the pathways from the MSigDB database v4.0 are randomly distributed at the top or bottom of the ranking. The significance threshold was set at p < 0.05.

### Statistical analysis

All analyses were performed with SPSS 18.0 software (SPSS Inc., Chicago, IL). All experiments were repeated at least three times to calculate the mean and standard deviation (SD). For comparisons, the Student’s *t* test, paired-samples t-test, and ANOVA analysis were performed as appropriate. Survival probabilities were evaluated using the Kaplan–Meier method, and differences were assessed using the log-rank test. p (two-sided) values less than 0.05 were statistically significant. Data were presented as the mean ± standard deviation (SD).

## Results

### KIF26B is overexpressed in HCC tissues and associated with poor prognosis

**Fig. 1:**
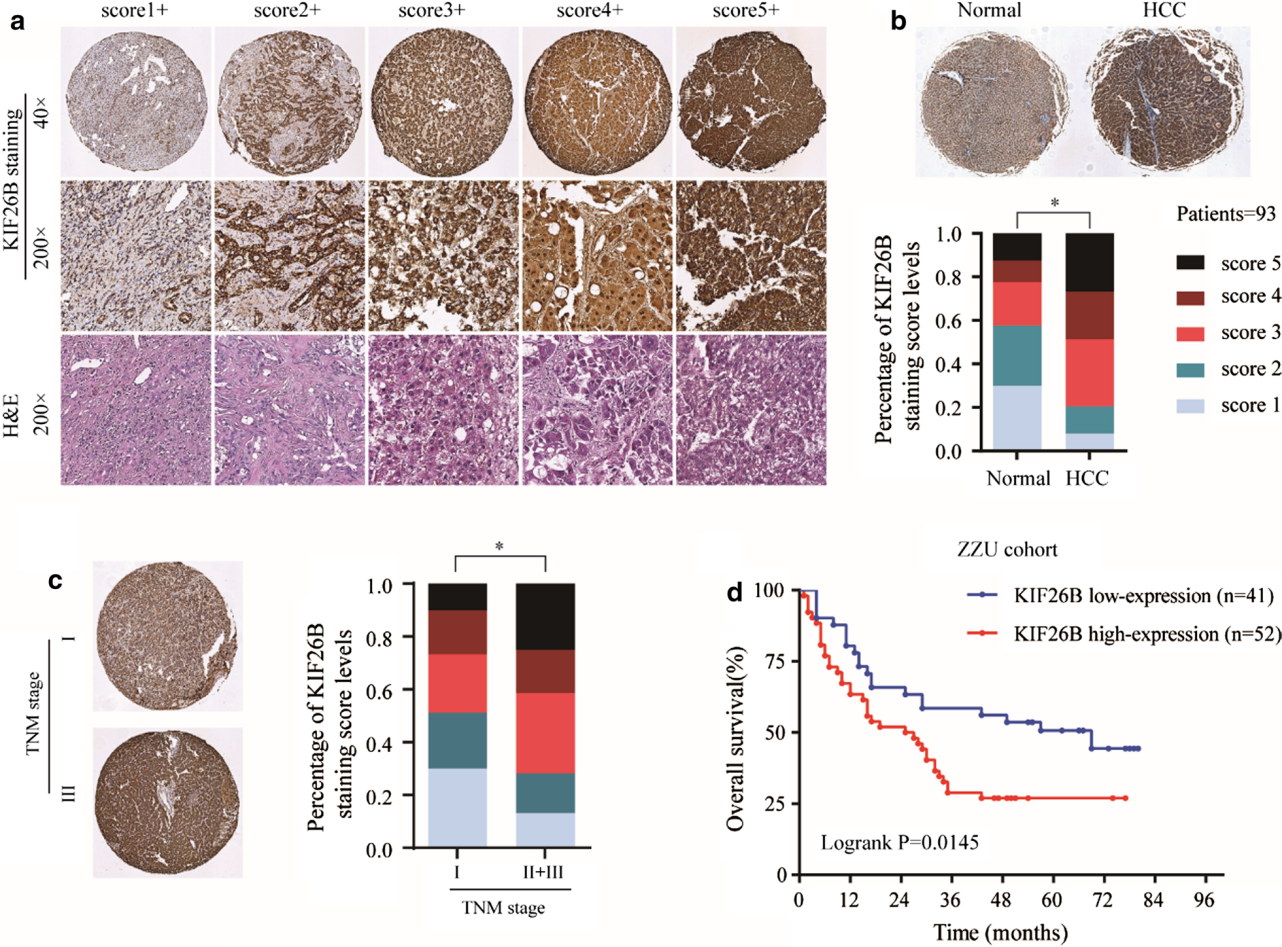
KIF26B was overexpressed in HCC tissues and associated with poor prognosis. **a**, **b** Immunohistochemistry results of 93 paired HCC tissues and adjacent normal tissues were scored according to the degree of staining according to 1–5, and the result of KIF26B expression level was quantified, *p < 0.05. **c** Expression of KIF26B between patients with TNM I stage and TNM II-III stage was compared and quantified, *p < 0.05. **d** Kaplan–Meier analysis was used to analyze correlation between KIF26B expression and overall survival of HCC patients

**Fig. 2:**
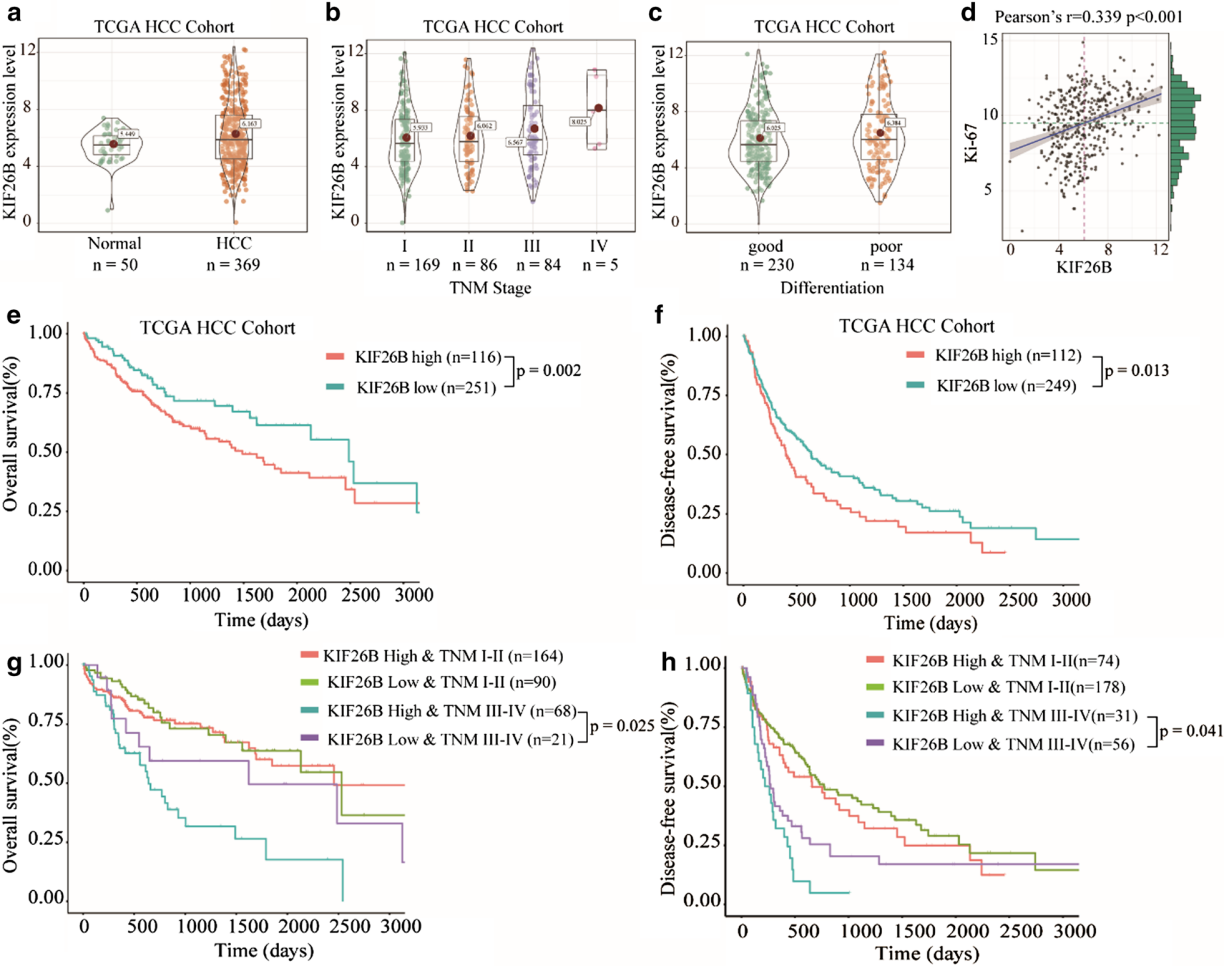
Expression and clinical relevance of KIF26B in TCGA cohort of HCC patients. **a** Expression of KIF26B in HCC tissues and normal tissues. **b**, **c** Expression of KIF26B in different TNM stage and cell differentiation of TCGA HCC cohort. **d** Relationship between expression level of KIF26B and Ki67 in TCGA HCC cohort. **e**, **f** Relationship between expression level of KIF26B and overall survival and disease-free survival of TCGA HCC patients. **g**, **h** Stratified analyses between TNM stages and KIF26B expression on overall survival and disease-free survival

To investigate function of KIF26B in progression of HCC, tissue microarrays (TMA) containing 93 paired HCC tissues and adjacent non-tumor tissues was used to detect KIF26B expression by IHC, the result showed that KIF26B was overexpressed in HCC tissues (Fig. 1a, b). Results of univariate analysis and Kaplan–Meier analysis revealed that high expression of KIF26B was correlated with late TNM stage, poor histological grade and poor overall survival (Fig. 2c, d, Additional file 1: Tables S1, S2). To further demonstrate our findings, a total of 369 HCC patients and 50 normal patients from The Cancer Genome Atlas (TCGA, https://tcga-data.nci.nih.gov/tcga/) database were used for study. The results showed that expression of KIF26B was higher in HCC patients and increases with tumor TNM stage, overexpression of KIF26B was associated with poor differentiation (Fig. 2a–c). We also found expression of KIF26B was positive correlated with Ki67 level (Fig. 2d). Kaplan–Meier analysis showed that high expression of KIF26B was associated with poor overall survival and disease-free survival (Fig. 2e, f). In addition, we divided patients into two groups including TNM I-II and TNM III-IV, and found high expression of KIF26B was associated poor prognosis in TNM III-IV group (Fig. 2g, h).

### Suppression of KIF26B inhibits proliferation and invasion of HCC cells in vitro

**Fig. 3:**
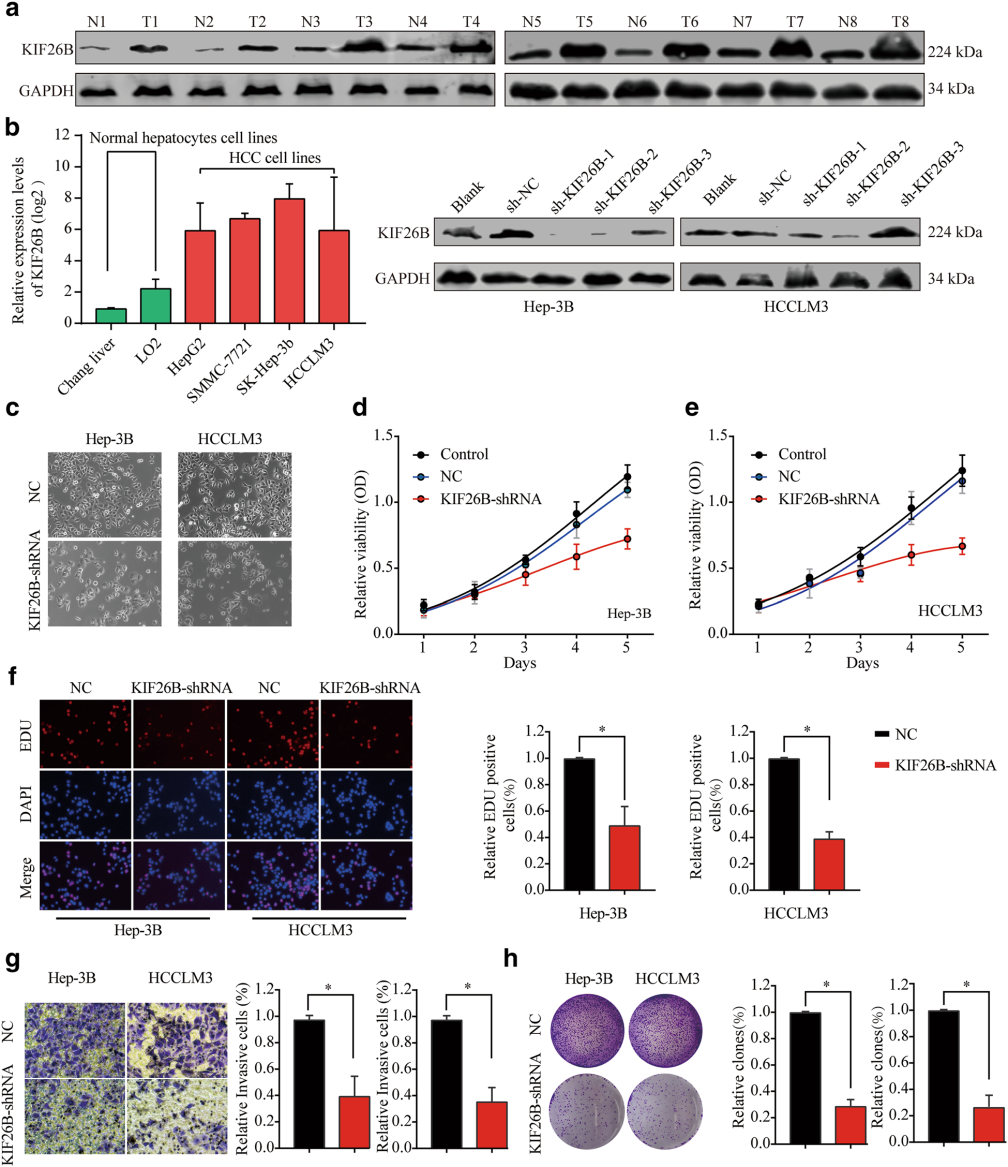
Suppression of KIF26B inhibits proliferation rate and invasion ability of HCC cells in vitro. **a** Expression difference of KIF26B between HCC tissues and adjacent non-tumor tissues was showed. **b** Protein level of KIF26B between normal hepatocytes cell lines and HCC cell lines was compared. And transfect efficiency of KIF26B shRNA in Hep-3B and HCC-LM3 cells was verified in protein level. **c**–**e** Cell viability of Hep-3B and HCC-LM3 cells under treatment of KIF26B shRNA and negative control in 24 h, 48 h and 72 h. **f**, **g** Cell proliferation rate and invasion ability of Hep-3B and HCC-LM3 cells under treatment of KIF26B shRNA and negative control. **h** Tumor formation ability of HCC cells with KIF26B shRNA and Negative control was compared in vitro. Mean ± SD, *p < 0.05, unpaired Student’s t test or one-way ANNOVA followed by multiple t test

To verify the above results, expression of KIF26B was detected by western blot among HCC tissues, adjacent non-tumor tissues, normal hepatocyte cell lines and HCC cell lines, and the results showed that KIF26B was overexpressed in HCC tissues and cell lines (Fig. 3a, b). Then, we selected Hep-3B and HCC-LM3 for further study, and KIF26B shRNA was used to suppress expression of KIF26B (Fig. 3b). We observed that suppression of KIF26B could significantly decrease cell viability and proliferation rate of HCC cells (Fig. 3c–f). The results of transwell assay showed that knockdown of KIF26B led to lower invasion ability of HCC cells (Fig. 3g). In addition, we found that suppression of KIF26B could inhibit the tumor formation ability of HCC cells in vitro (Fig. 3h). Taken together, these loss of function experiments showed that KIF26B was critical and important for proliferation and invasion of HCC cells.

### Suppression of KIF26B inhibits tumor formation ability of HCC cells in vivo

**Fig. 4:**
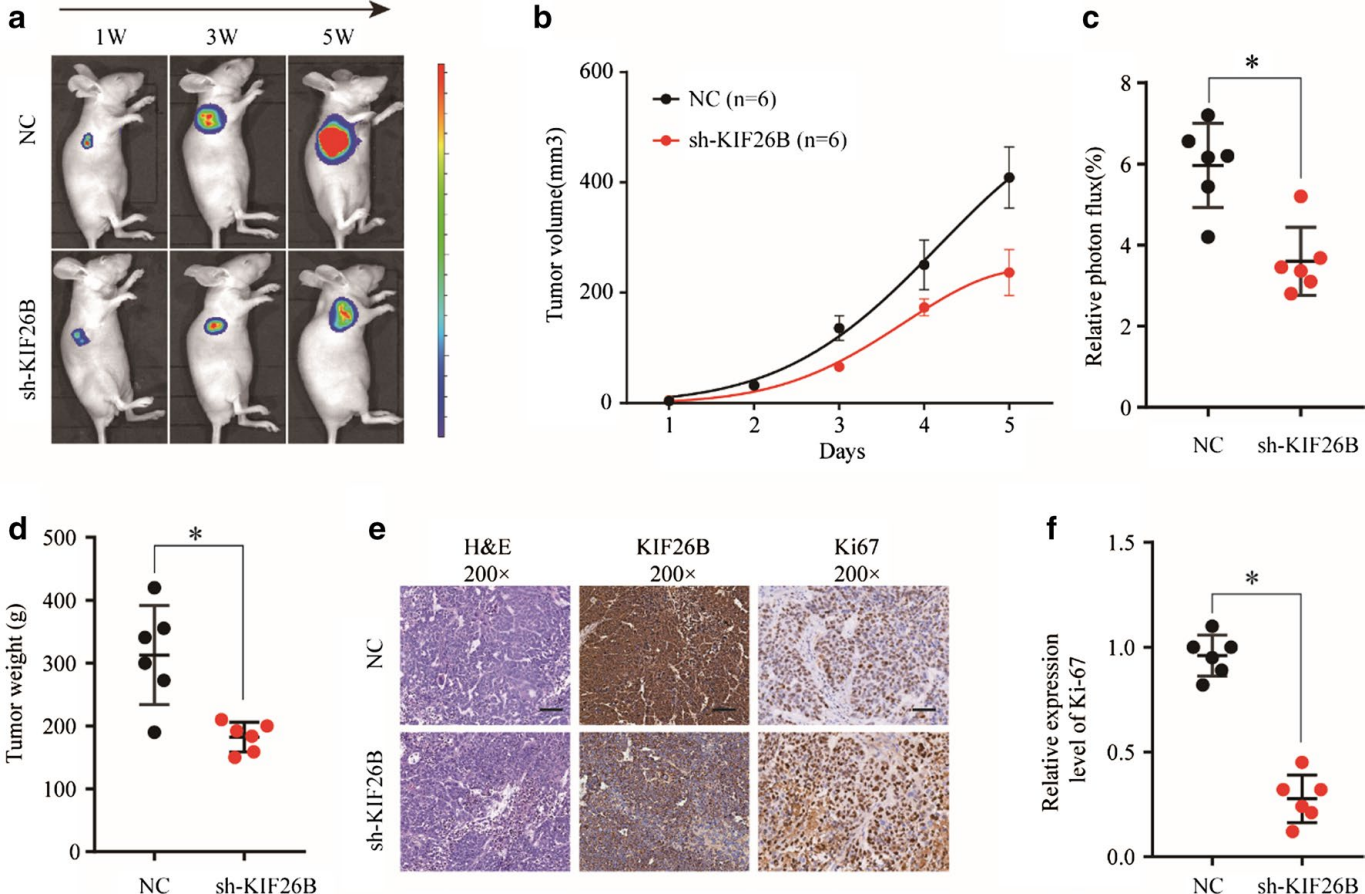
Suppression of KIF26B inhibits tumor formation ability of HCC cells in vivo. **a**–**d** The in vivo effect of KIF26B was evaluated in xenograft mouse models bearing tumors originating from HCC-LM3 cells via tumor volume, photon flux and tumor weight; n = 6 per group. **e**, **f** Expression of KIF26B and Ki67 were detected between KIF26B shRNA and negative control groups in node mouse tumor tissues

To further demonstrate function of KIF26B in vivo, we constructed KIF26B knockdown stable cell line and conducted subcutaneous tumor formation in nude mice. The results showed that the shKIF26B group had smaller tumor volume, weaker relative photon flux and lighter tumor weight than negative control group (Fig. 4a–d). Then, we detected expression of Ki67 in nude mouse tumor tissue by IHC, and found that expression of Ki67 was significantly decreased under condition of KIF26B knockdown (Fig. 4e, f). Thus, we demonstrated that suppression of KIF26B could affect tumor formation ability of HCC cells in vivo.

### KIF26B regulates proliferation and invasion of HCC cells through PI3K/AKT pathway

**Fig. 5:**
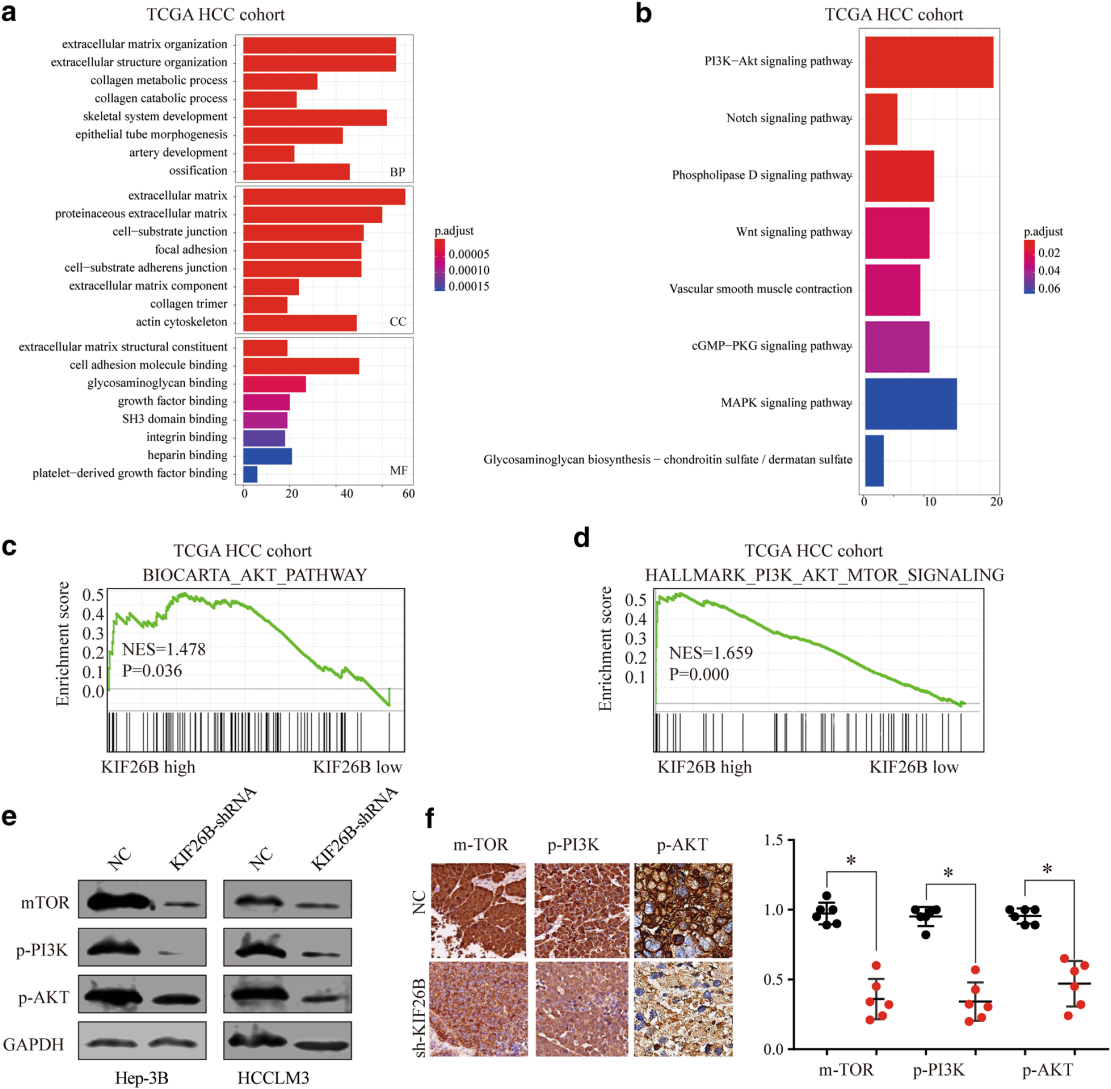
KIF26B regulates proliferation and invasion of HCC cells through PI3K/AKT pathway. **a**, **b** The GO and KEGG enrichment analysis was conducted in TCGA HCC cohort between KIF26B high expression and KIF26B low expression. **c**, **d** The Gene Set Enrichment Analysis plot indicated a significant correlation between KIF26B and PI3K/AKT pathway. **e**, **f** Expression change of m-TOR, p-AKT and p-PI3K in HCC cells and xenograft mouse models bearing tumors under condition of KIF26B suppression

To investigate the mechanism of KIF26B on the function of HCC cells, we identified the top 800 genes which were the highest correlation with KIF26B expression in the TCGA liver cancer database for KEGG and GO analysis, and the result showed that PI3K/AKT signaling pathway was the significant enrichment (Fig. 5a, b). And the Gene Set Enrichment Analysis (GSEA) of the TCGA-LIHC data set revealed high expression of KIF26B was associated with activation of AKT pathway (Fig. 5c, d).

Then we detected expression change of m-TOR, p-PI3K and p-AKT in HCC cells with KIF26B suppression, the result showed that these proteins were significantly decreased under condition of KIF26B knockdown (Fig. 5e). In addition, we detected expression of m-TOR, p-PI3K and p-AKT in nude mouse tumor tissues by IHC, and we found that all these protein levels were lower in shKIF26B group than negative control group (Fig. 5f). Thus, we proved that KIF26B might affect proliferation and invasion of HCC cells via PI3K/AKT signaling pathway.

### KIF26B is targeting regulated by miR-450b-5p in HCC cells

**Fig. 6:**
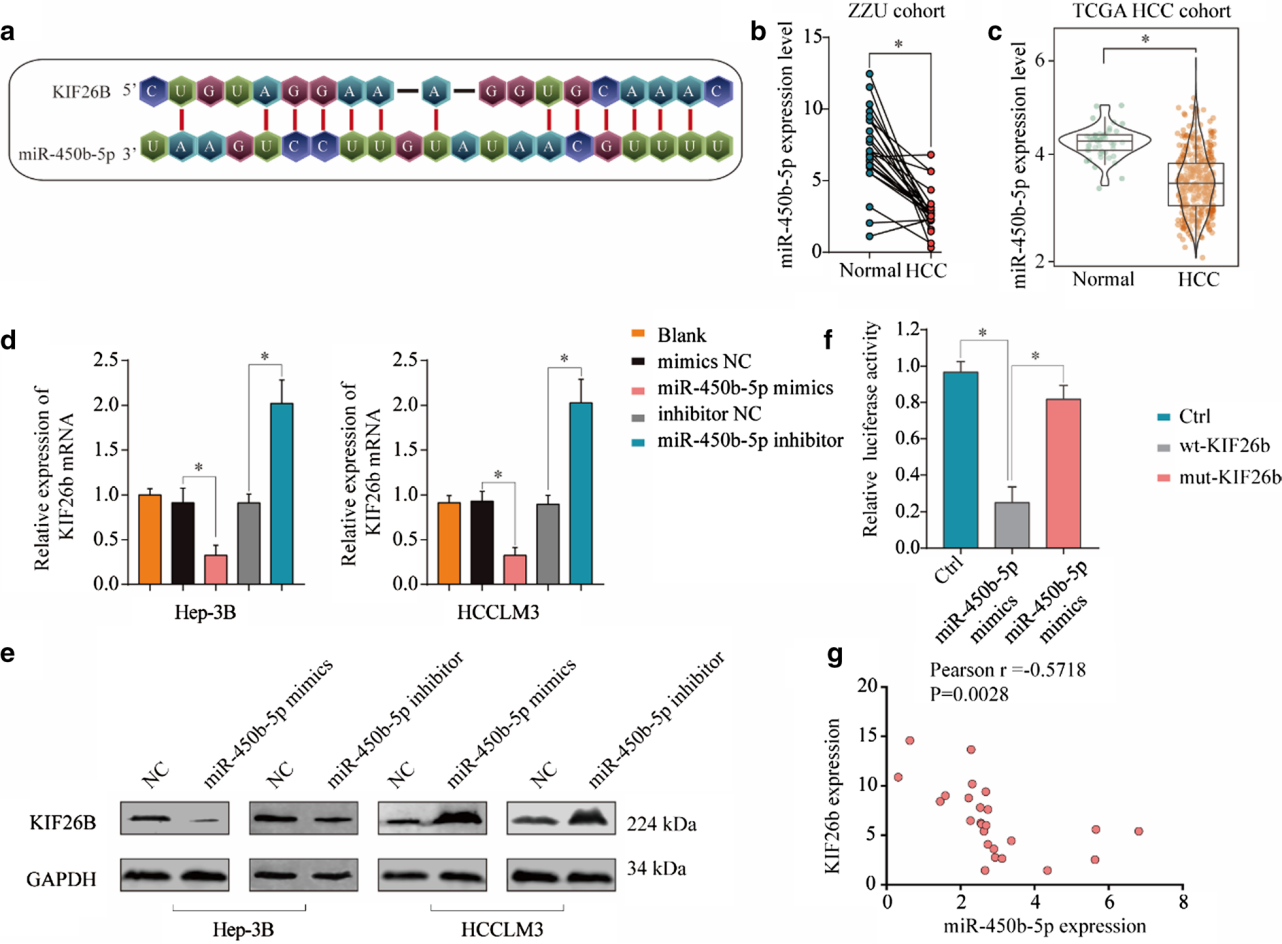
KIF26B is targeting regulated by miR-450b-5p in HCC cells. **a** miR-450b-5p could be specific complementary pairing with 3′UTR sequence of KIF26B. **b**, **c** Expression difference of miR-450b-5p between HCC tissues and adjacent non-tumor tissues in our hospital HCC cohort and TCGA HCC cohort. **d**, **e** Protein and mRNA change of KIF26B in HCC cells under treatment of miR-450b-5p mimics, inhibitor and negative control. **f** Relative luciferase activity between KIF26B wild type and mutant type based on a dual-luciferase reporter assay. Values represent the mean ± SD (n = 5, each). **g** Relationship between KIF26B expression and miR-450b-5p expression in HCC tissues. *p < 0.05

Our study found that miR-450b-5p could be complementary pairing with 3′UTR sequence of KIF26B, and observed that miR-450b-5p was downregulated in HCC tissues (Fig. 6a–c). Then, we observed that miR-450b-5p mimics could inhibit both mRNA and protein levels of KIF26B in HCC cells while the results of miR-450b-5p inhibitor were the opposite (Fig. 6d, e). To further prove miR-450b-5p could target regulate expression of KIF26B, Luciferase reporter assay was conducted and we observed that luciferase activity of wild-type group was significantly decreased under condition of miRNA-450b-5p mimics, such results showed the specificity of the interaction between miRNA-450b-5p and the 3′UTR sequence of KIF26B (Fig. 6f). In addition, correlation analysis unveiled that the expression of KIF26B was negative correlated with miR-450b-5p expression in HCC tissues (Pearson r = − 0.5718)(Fig. 6g). Taken together, our study demonstrated that KIF26B was downstream target gene of miR-450b-5p in HCC cells.

### Overexpression of KIF26B could reverse tumor suppressive effect of miR-450b-5p in HCC cells

**Fig. 7:**
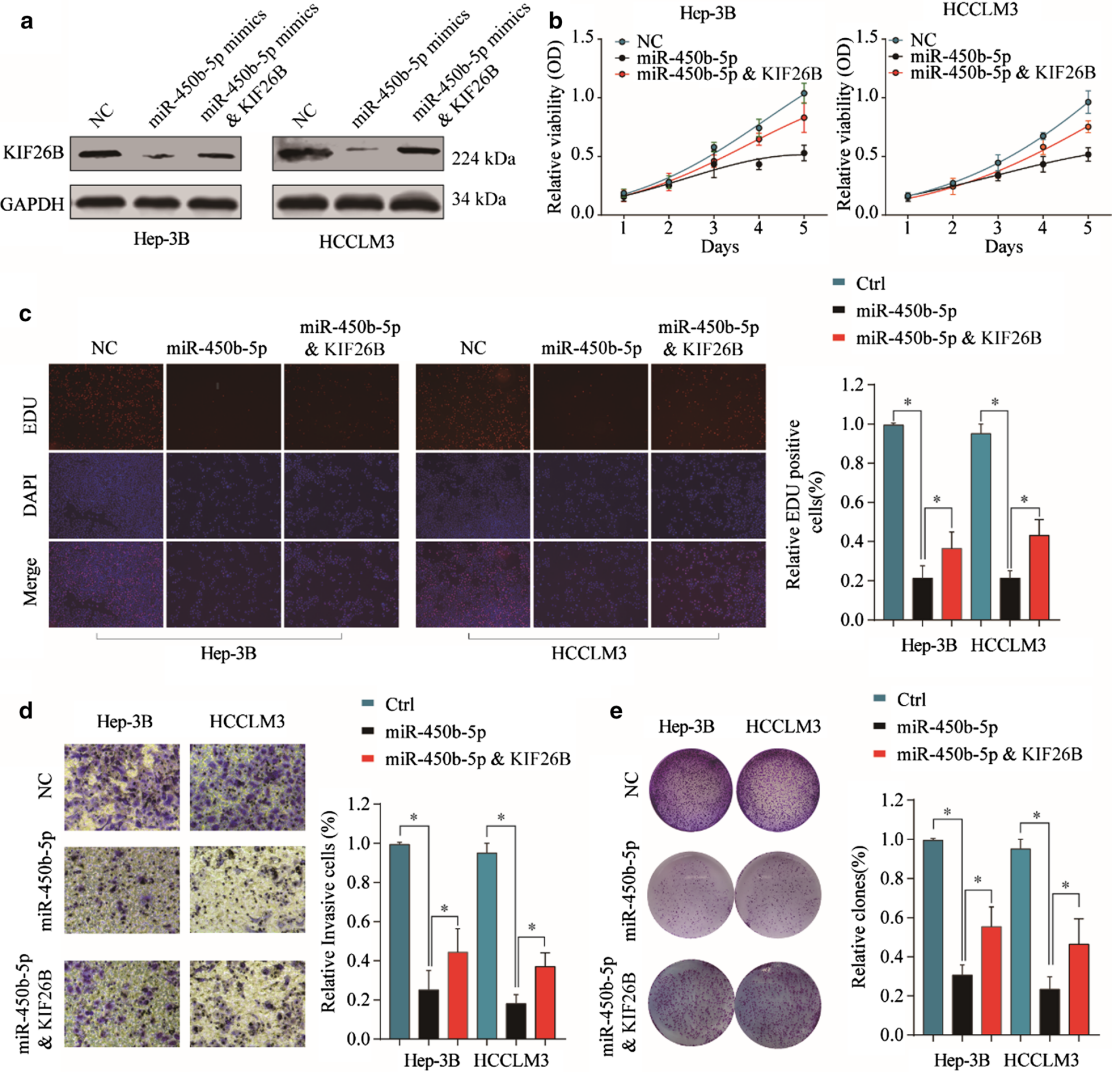
Overexpression of KIF26B could reverse tumor suppressive effect of miR-450b-5p in HCC cells. **a** KIF26B expression under condition of miR-450b-5p mimics, miR-450b-5p mimics and KIF26B or negative control. **b** Cell viability of Hep-3B and HCC-LM3 cells under treatment of miR-450b-5p mimics, miR-450b-5p mimics and KIF26B and negative control in 24 h, 48 h and 72 h. **c**, **d** Cell proliferation rate and invasion ability of Hep-3B and HCC-LM3 cells under treatment of miR-450b-5p mimics, miR-450b-5p mimics and KIF26B and negative control. **e** Tumor formation ability of HCC cells with miR-450b-5p mimics, miR-450b-5p mimics and KIF26B and negative control was compared in vitro. Mean ± SD, *p < 0.05, unpaired Student’s t test or one-way ANNOVA followed by multiple t test

To investigate whether overexpression of KIF26B could reverse tumor suppressive effect of miR-450b-5p in HCC cells, we constructed KIF26B overexpression plasmid and observed that such plasmid could almost reverse KIF26B suppression effect of miR-450b-5p in HCC cells (Fig. 7a). Then we conducted cell function experiments to investigate the efficiency of KIF26B overexpression plasmid in HCC cells under treatment of miR-450b-5p mimics. The results showed that miR-450b-5p could significantly inhibit cell viability, proliferation rate, cell invasion ability and colony formation ability of HCC cells, while KIF26B overexpression plasmid could partially reverse the tumor suppressive effect of miR-450b-5p in HCC cells (Fig. 7b–e). Thus, our study demonstrated that overexpression of KIF26B could partially reverse tumor suppressive effect of miR-450b-5p in HCC cells.

### Negative correlation between KIF26B and miR-450b-5p is associated with prognosis of HCC patients

**Fig. 8:**
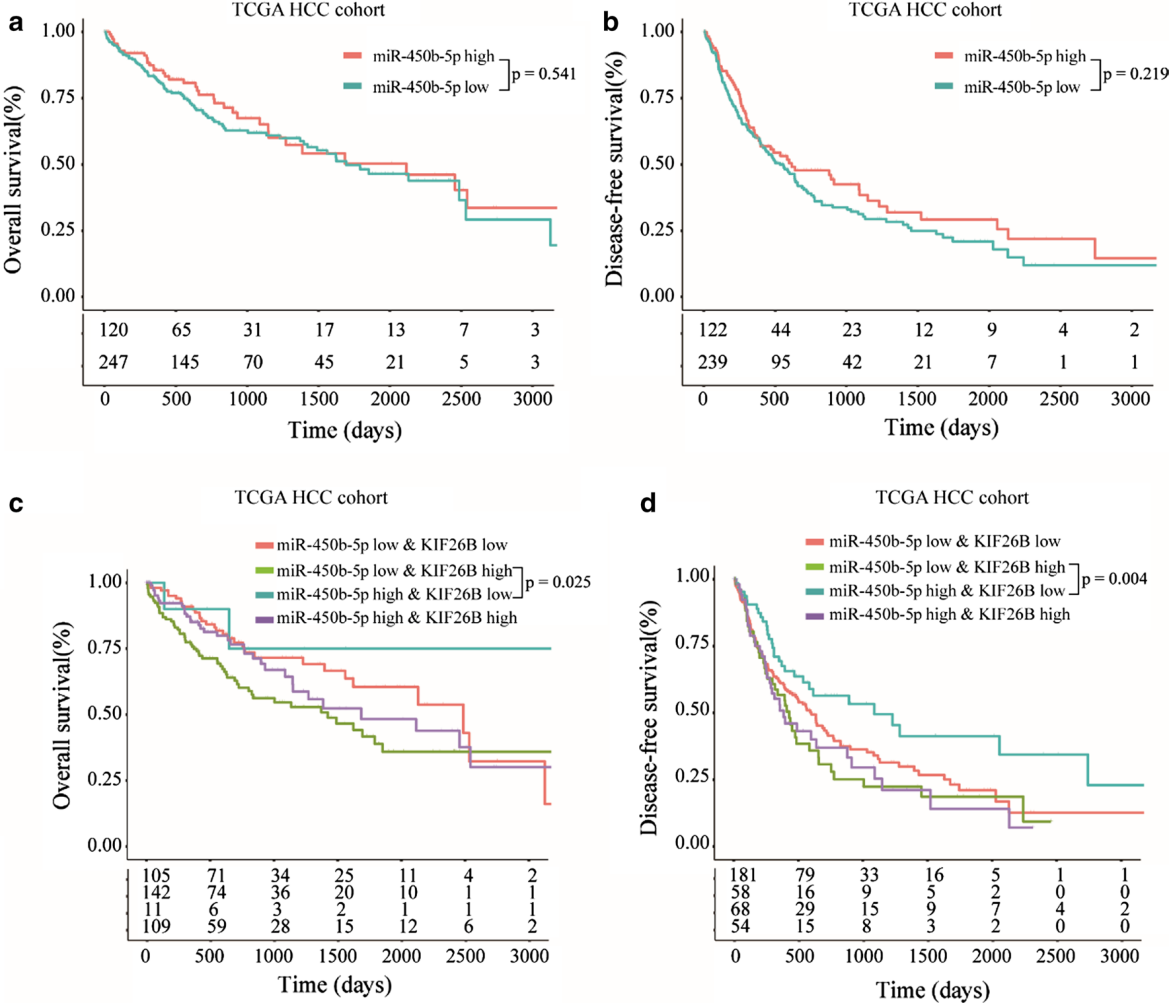
Negative correlation between KIF26B and miR-450b-5p is associated with prognosis of HCC patients. **a**, **b** Relationship between expression level of miR-450b-5p and overall survival and disease-free survival of TCGA HCC patients. **c**, **d** Overall survival and disease-free survival were compared among HCC patients with miR-450b-5p^low^KIF26B^low^, miR-450b-5p^low^KIF26B^high^, miR-450b-5p^high^KIF26B^low^ and miR-450b-5p^high^KIF26B^high^

To investigate relationship between miR-450b-5p expression and prognosis of HCC patients, TCGA HCC cohort was used and the result of Kaplan–Meier analysis showed that miR-450b-5p level was no correlation with overall survival and disease-free survival of HCC patients (Fig. 8a, b). However, when we divided these patients into four groups, including miR-450b-5p^low^KIF26B^low^, miR-450b-5p^low^KIF26B^high^, miR-450b-5p^high^KIF26B^low^ and miR-450b-5p^high^KIF26B^high^, we found that HCC patients who with miR-450b-5p^low^KIF26B^high^ always associated worse overall survival and disease-free survival than those with miR-450b-5p^high^KIF26B^low^ (Fig. 8c, d). Collectively, these data indicated that negative correlation between KIF26B and miR-450b-5p could be considered as a prognosis marker for HCC.

## Discussion

Initiation and progression of hepatocellular carcinoma is complex processes involving changes in gene expression, signaling pathways, and epigenetics. Abnormal expression of the core genes often results in dysregulated cell growth, differentiation, apoptosis, and migration [13]. Therefore, investigating for the abnormally expressed core genes is important for developing new target for HCC treatment. Low early diagnosis rate and postoperative recurrence of liver cancer have been the two major factors affecting the prognosis of patients with liver cancer [2, 3]. Therefore, it is important to investigate new therapeutic targets which could act as a potential biomarker for the early diagnosis and prognosis of patients with HCC.

The kinesin superfamily proteins (KIFs) are molecular motor proteins which correlated with microtubule binding and ATPase activities [14]. Recent studies have proved that KIF26B acted as critical role in the regulation of many physiological events, including brain function [15], developmental patterning [4], and proliferation and migration of solid tumors [5–8, 16, 17]. Other study demonstrated that KIF26B played important role in multi-drug resistance in osteosarcoma [9]. However, there is limited evidence of the function of KIF26B during progression of HCC. In this study, we found KIF26B was overexpressed in HCC tissues, high expression of KIF26B was correlated with later TNM stage, poor tumor differentiation and prognosis. Suppression of KIF26B could inhibit proliferation rate and invasion ability of HCC cells in vitro, and affect tumor formation ability both in vitro and in vivo.

To further explore the molecular mechanism of knockdown of KIF26B on the proliferation and invasion of HCC cells, we used the KEGG and GO analysis through TCGA database to find the signaling pathway which was the most relevant to KIF26B expression levels. We found PI3K/AKT signaling pathway may regulated by KIF26B, and we proved that suppression of KIF26B could decrease expression of m-TOR, p-AKT and p-PI3K both in vitro and in vivo. Constitutive activation of the PI3K/AKT/mTOR signaling pathway acted as critical role in the progression of HCC, including cell proliferation, migration and invasion, angiogenesis and distant metastasis [18]. Thus, we proved that suppression of KIF26B could inhibit proliferation, invasion and tumor formation ability of HCC cells through regulating activation of PI3K/AKT signaling pathway.

MiRNAs are noncoding RNAs with ~ 22 nucleotides which play vital role in regulating gene expression via inhibiting posttranscriptional translation of target mRNAs [19]. To investigate upstream regulatory factor of KIF26B, we found miR-450b-5p could be specific complementary pairing with 3′UTR sequence of KIF26B mRNA. MiR-450b-5p has been reported associated with progression of several solid tumors, including colorectal cancer [20], lung adenocarcinoma [21], rectal cancer [22], prostate cancer [23], rhabdomyosarcoma [24]. However, the function of miR-450b-5p in progression of HCC was unclear. In this study, we found miR-450b-5p was negative correlated with KIF26B expression in HCC tissues, and miR-450b-5p mimics and inhibitor could significantly affect KIF26B expression in HCC cells. Furthermore, we observed that miR-450b-5p could obviously inhibit cell viability, proliferation rate, invasion ability and tumor formation ability of HCC cells, while overexpression KIF26B could partially reverse tumor suppression function of miR-450b-5p. In addition, we found HCC patients who with miR-450b-5p^low^KIF26B^high^ always associated poor prognosis than those with miR-450b-5p^high^KIF26B^low^. Thus, our study demonstrated that KIF26B was one of the target genes of miR-450b-5p, low expression of miR-450b-5p could induce overexpression of KIF26B, and then promote activation of PI3K/AKT pathway.

## Conclusion

Our study found that KIF26B was overexpressed in HCC tissues and was an independent risk factor for prognosis of HCC patients. Suppression of KIF26B could affecting progression of HCC via inhibiting activation of PI3K/AKT pathway. KIF26B was target regulated by miR-450b-5p, and loss of miR-450b-5p maybe one reason for overexpression of KIF26B in HCC tissues. This discovery might provide new biomarker and therapeutic target for early diagnosis and treatment of HCC. However, further studies were needed to demonstrated the deeper molecular mechanism of KIF26B regulating activation of PI3K/AKT pathway.

## Additional file

**Additional file 1: Table S1.** The relationship between KIF26b expression and clinicopathological features of ZZU HCC cohort. **Table S2.** Univariate and multivariate analyses of overall survival of ZZU HCC cohort.

## Data Availability

All the data and material could be traced from the paper we have published before.

## Abbreviations

KIF26B: kinesin family member 26B
HCC: hepatocellular carcinoma
OS: overall survival
TMA: tissue microarrays
qRT-PCR: quantitative real-time PCR
CCK-8: cell counting kit-8
3′UTR: 3′ untranslated region
GSEA: the gene set enrichment analysis
SD: standard deviation
