# Supplementary material for: miR-450b-5p loss mediated KIF26B activation promoted hepatocellular carcinoma progression by activating PI3K/AKT pathway
Source: Cancer Cell Int. 2019 Jul 31;19:205. doi: 10.1186/s12935-019-0923-x (PMC6670205; doi:10.1186/s12935-019-0923-x)
Supplement: Supplementary file 1 — Additional file 1: Table S1. The relationship between KIF26b expression and clinicopathological features of ZZU HCC cohort. Table S2. Univariate and multivariate analyses of overall survival of ZZU HCC cohort. [file 12935_2019_923_MOESM1_ESM.docx]

|  | | Clinicopathological features | No. of  cases | KIF26b expression | | P |
| --- | --- | --- | --- | --- | --- | --- |
|  |  |  |  | Low (n=44) | High (n=49) |  |
| Age(years) | <median | | 41 | 19 | 22 | 0.867 |
|  | >median | | 52 | 25 | 27 |  |
| Gender | Male | | 46 | 22 | 24 | 0.921 |
|  | Female | | 47 | 22 | 25 |  |
| Size | <5cm | | 42 | 20 | 22 | 0.957 |
|  | >5cm | | 51 | 24 | 27 |  |
| TNM stage | Stage I and II | | 45 | 27 | 18 | 0.017^*^ |
|  | Stage III and IV | | 48 | 17 | 31 |  |
| Histological grade | grade 1-2 | | 51 | 31 | 20 | 0.004^*^ |
|  | grade 3-4 | | 42 | 13 | 29 |  |

**Additional file 1: Table S1. The relationship between KIF26b expression and clinicopathological features of ZZU HCC cohort**

Abbreviations: TNM=tumor-node-metastasis. ^*^*p*<0.05, ^**^ *p* <0.001

**Additional file 1: Table S2. Univariate and multivariate analyses of overall survival of ZZU HCC cohort**

|  | Clinicopathological features | Univariate analyses | | | Multivariate analyses | | |
| --- | --- | --- | --- | --- | --- | --- | --- |
|  |  | HR | 95%（Cl） | *P* value | HR | 95%（Cl） | *P* value |
| Age(years) | <median | 1.000 | 0.763-1.201 | 0.465 |  |  |  |
|  | >median | 0.745 |  |  |  |  |  |
| Gender | Male | 1.000 | 0.255-0.915 | 0.364 |  |  |  |
|  | Female | 0.657 |  |  |  |  |  |
| Tumor size | <5cm | 1.000 | 1.122-2.517 | 0.040^*^ | 1.000 | 0.914-2.106 | 0.095 |
|  | >5cm | 1.751 |  |  | 1.461 |  |  |
| TNM stage | Stage I and II | 1.000 | 2.141-5.967 | 0.015^*^ | 1.000 | 2.331-4.876 | 0.023^*^ |
|  | Stage III and IV | 3.958 |  |  | 3.195 |  |  |
| Histological grade | grade 1-2 | 1.000 | 1.184-2.249 | 0.174 |  |  |  |
|  | grade 3-4 | 1.698 |  |  |  |  |  |
| KIF26b expression | Low | 1.000 | 1.822-5.941 | 0.029^**^ | 1.000 | 1.751-3.159 | 0.039^*^ |
|  | High | 2.987 |  |  | 2.487 |  |  |

Abbreviations: TNM=tumor-node-metastasis; HR=hazard ratio; CI= confidential interval. ^*^ *p* <0.05, ^**^ *p* <0.001
